# Relevance of Mathematical Optimization as a Tool for Diet Modeling in the Development of Food-Based Dietary Recommendations in Sub-Saharan Africa: A Scoping Review

**DOI:** 10.1016/j.advnut.2025.100480

**Published:** 2025-07-11

**Authors:** Sakiko Shiratori, MG Dilini Abeysekara

**Affiliations:** 1 Information and Public Relations Office, Japan International Research Center for Agricultural Sciences (JIRCAS), Tsukuba, Ibaraki, Japan; 2 Graduate School of Agricultural and Life Sciences, The University of Tokyo, Yayoi, Tokyo, Japan

**Keywords:** food-based dietary recommendations, linear programming, diet optimization, sub-Saharan Africa, scoping review

## Abstract

This study aimed to understand the role of mathematical programming in the development of food-based dietary recommendations (FBRs) in sub-Saharan Africa (SSA), identify current limitations, and highlight opportunities for advancing evidence-based dietary interventions. Following the Preferred Reporting Items for Systematic Reviews and Meta-Analyses Extension for Scoping Reviews, a systematic search from January 2000 to May 2024 identified 97 relevant studies. Among these, 30 studies spanning 12 SSA countries (of 48 countries and territories in SSA) met the inclusion criteria. The reviewed studies leveraged linear programming (LP) or extensions of LP (i.e., linear goal programming) to formulate FBRs by optimizing current dietary patterns to meet nutritional needs and gaps (*n* = 24), developing nutritionally and regionally optimized and cost-minimized food baskets (*n* = 4), and describing the use of LP as a method for designing population-specific food-based dietary guidelines (*n* = 2). The primary goal of the reviewed studies is to develop nutritionally adequate and economically affordable food patterns, rather than to address multiple chronic nutrition-related conditions simultaneously, reflecting the distinct priorities of diet modeling in low-resource settings compared with those of resource-rich contexts. The formulated FBRs and optimized diets are often defined for specific demographic groups, with a limited geographic scope reflecting regional priorities. Diets can be optimized both nutritionally and economically by prioritizing locally available food groups and items; however, in some cases, additional supplementation and or inclusion of rarely consumed nutrient-dense foods may be necessary. Mathematical optimization, particularly LP, is a valuable tool for addressing dietary challenges and developing evidence-based, context-specific FBRs. Its use is facilitated by the availability of user-friendly software. However, its successful application requires high-quality input data, consideration of behavioral and practical aspects, and interdisciplinary collaboration. High-quality input data and incorporating sociocultural contexts are critical for leveraging mathematical optimization to inform inclusive and effective dietary recommendations in SSA.

**Statement of significance**

This scoping review has synthesized and mapped the application of mathematical modeling in the formulation of context- and population-specific food-based recommendations. Mathematical optimization can yield the most viable dietary options. However, in resource-limited settings, the primary focus tends to be on enhancing dietary choices to ensure nutritional benefits and economic feasibility, with less emphasis placed on addressing nutrition-related chronic health conditions, particularly when compared with that in resource-rich environments.

## Introduction

Sub-Saharan Africa (SSA) is experiencing a severe food insecurity and nutrition crisis, characterized by the triple burden of malnutrition, namely, the coexistence of undernourishment, micronutrient deficiencies, and overnutrition. These concerns are closely related to diet, which is driven by various factors, such as agricultural production, marketing strategies, and lifestyle changes. For example, the consumption of affordable processed foods rich in energy, fat, and salt yet lacking in nutrient quality [1] has increased. It is necessary to initiate a dietary shift toward a healthy diet. The high burden of malnutrition in all forms, combined with rapid population growth and limited availability of robust data and analysis compared with other world regions, led us to focus on SSA in this scoping review.

Various healthy diets worldwide show that there is no singular approach to maintaining a healthy diet, nor a specific pattern that can be universally recommended to individuals. Dietary habits are deeply rooted in local and regional traditions and are shaped by a complex interplay of social and economic factors, including food prices, income, individual preferences, beliefs, as well as cultural, geographical, and environmental influences [2,3].This has led to the need for region-specific dietary recommendations that draw on comprehensive evidence about local food environments and incorporate stakeholder perspectives [4]. To guide populations effectively, many governments develop food-based dietary guidelines (FBDGs), which outline the key components of a healthy diet tailored to national contexts. FBDGs aim to encourage healthy eating habits and form the foundation of public health policies related to food and offer practical recommendations for achieving adequate nutrient intake and promoting dietary diversity across various food groups. Adherence to national dietary guidelines is associated with improved micronutrient adequacy and a reduced risk of diet-related chronic diseases, including diabetes, obesity, hypertension, cardiovascular diseases, and certain types of cancer [5].

Such interventions have been increasingly prioritized in many domestic policy agendas in the SSA region [6]. However, diets that align with official national FBDGs are deemed unaffordable for ∼72% of the SSA population as of 2022 [7]. As nutritious diets are currently beyond the reach of many low-income consumers, many studies have emphasized the importance of reducing the prices of nutrient-dense foods [8], improving income levels [9] and promoting dietary diversity through the use of locally produced foods to address nutrient supply gaps [10]. Efforts to comprehend and address the issue of malnutrition in SSA have demonstrated that dietary guidelines can be effective, provided if only that essential factor such as practicality, affordability, acceptability, and the diverse influences on dietary choices are considered.

Diet optimization achieved through mathematical approaches translates nutritional requirements, which are expressed as recommended daily intakes, into food selections, although considering various food-related factors such as consumption habits and prices. In the past, optimal diets were developed through expert consultations using a trial-and-error approach. At present, more efficient processes are enabled by rigorous mathematical modeling [11]. A high-profile focus has been placed upon evidence-based mathematical modeling approaches in developing population-tailored FBDGs to ensure that guidelines are developed transparently, free from explicit bias, and based on the best available evidence.

Diet modeling using linear programming (LP), a widely applied mathematical optimization technique, aims to identify the optimal combination of foods (decision variables) that either minimize or maximize a linear objective function, subject to a set of linear constraints [2,11,12]. In dietary applications, objective functions commonly focus on minimizing deviations from current dietary patterns, reflecting the assumption that individuals prefer diets similar to their existing habits, or minimizing total diet cost although meeting nutrient requirements. The choice of objective function depends on the study’s focus and data availability; when reliable price data are lacking, cost minimization is often replaced with minimizing standardized differences between observed and recommended food intakes. As such deviations introduce nonlinearity, they must be reformulated into linear terms to ensure a global optimum, typically through linear goal programming—an extension of LP that accommodates multiple goals [2]. Decision variables generally represent quantities of foods or food groups, whereas constraints are imposed to ensure nutritional adequacy, cultural acceptability, and practical feasibility.

LP has been extensively discussed as useful tools for developing and testing [12] and integrating various dimensions of FBDGs [13,14]. In some instances, such approaches have been used to inform the development of healthy eating patterns at the national level [[15], [16], [17], [18], [19], [20]] for specific age groups [21] and to test the nutrient relevance of FBDGs [22]. Several systematic reviews have documented the development process of the use of mathematical approaches in modeling diets [3,[23], [24], [25], [26]].

Despite its significance, diet optimization and modeling have primarily been used by high-income countries and not widely incorporated into the FBDGs of many low-income and middle-income countries [20]. However, recent evidence indicates that, in SSA, FBDGs have been underpinned by mathematical modeling, as highlighted by the recent dietary guidelines developed in Benin [17], Ethiopia [18], and Ghana [20]. Moreover, an emerging area of research has focused on developing context-specific food-based recommendations (FBRs) to promote the consumption of locally available nutrient-dense foods to achieve recommended nutritional targets [[27], [28], [29]].

A systematic scoping review of the literature related to approaches used in developing FBRs in SSA, where necessary data and resources are often lacking, could provide a comprehensive overview of the available evidence. This scoping review aimed to address this research gap by documenting the diet modeling approaches used in formulating diets that are optimized nutritionally and economically within the SSA context. Specifically, the review aimed to *1*) summarize the application of mathematical optimization in diet formulation, including its goals and the key components of optimization analysis; *2*) describe the outcomes of such modeling, including locally appropriate optimized diets and/or FBRs describe the final outputs; and *3*) explore the potential of mathematical optimization as a tool for modeling diets in developing FBRs in SSA while identifying limitations and opportunities for further development. The findings are expected to inform policymakers by highlighting the role of mathematically optimized food pattern modeling in guiding food choice decisions and shaping effective nutrition policies.

## Methods

A scoping review was conducted following the PRISMA—Extension for Scoping Reviews [30] to ensure a robust and replicable review process. A scoping review is important for synthesizing knowledge from a wide range of literature that has not yet been reviewed [30,31]. In contrast to systematic reviews, it maps fundamental concepts that form the basis of a research area [32].

Following Arksey and O’Malley [32], a 2-step strategy was used to identify relevant studies. First, we conducted a systematic literature search of PubMed and Web of Science for articles published between January 2000 and May 2024, selecting studies based on their focus on nutritional and health outcomes. Briefly, we combined search terms in sets as follows: *1*) dietary recommendations or recommended diet (i.e., FBRs, healthy diet, sustainable diet, optimal diet, and balanced diet); *2*) diet modeling (e.g., diet optimization, mathematical programming, LP, quadratic programming, and goal programming); and *3*) SSA countries (i.e., sub-Sahara, SSA, Sahel, and 48 individual countries in SSA as defined by the World Bank) [33]. These search sets were intended to capture the breadth of the available literature. The full search strategy used in Web of Science is detailed in Supplemental Material. In the second stage, we conducted an additional search using the reference lists of included article that met the inclusion criteria, followed by a manual search of key journals, relevant organizations, and conferences.

We borrowed the eligibility criteria for documents included in this review from the population–exposure–outcome–study design (PEOS) framework, which is commonly used in systematic analyses of the correlation between exposure and health outcomes [34]. We adopted the PEOS design framework by relating the diet modeling approach to the exposure, with the healthy diet being viewed as the associated outcome. Original peer-reviewed published articles and other scholarly communications were included if they met the following criteria: *1*) assessed and reported a healthy diet, nutritious diet, sustainable diet, or dietary recommendations using a mathematical approach and *2*) for any community in SSA countries. We excluded articles that did not meet the following criteria: *1*) mathematical optimization was not involved in assessing and providing dietary recommendations and *2*) the studies did not include SSA countries. We excluded studies on very defined populations (i.e., cancer patients) [35], even if those studies were from SSA countries; and studies on specific foods as opposed to diets (i.e., ready-to-use therapeutic foods) [36,37], clinical diets [38], and porridge mix [39]. Table 1 [5,13,40] details the eligibility criteria used to inform the inclusion and exclusion decisions for the documents.

**TABLE 1:** Eligibility criteria for included documents according to the population–exposure–outcome–study (PEOS) design framework.

	Inclusion criteria	Exclusion criteria	Rationale
Population	Individuals, households, and communities in SSA countries Studies conducted in multiple countries were included if ≥1 of the countries studied is within the SSA region^1^	Individuals, households, and communities outside SSA countries, and highly specified populations (e.g., cancer patients or individuals with AIDS) within SSA	The high burden of malnutrition in all forms, combined with rapid population growth and limited availability of robust data and analysis compared with other world regions, led us to focus on SSA in this scoping review.
Exposure	Studies focusing on mathematical modeling approaches (e.g., linear programming) in diet modeling and the development of dietary recommendations	Studies focusing on developing protocols, tools, or intervention designs	Diet modeling is a valuable tool in identifying complex nutritional problems. Evidence-based mathematical modeling is gaining significant attention in the development of dietary recommendations, as it ensures that guidelines are developed transparently, free from overt bias, and based on the best available evidence [13].
Outcome	Population-specific healthy diets, nutritious diets, and sustainable diets or dietary recommendations catering to the nutritional needs of specific population groups [e.g., pregnant and lactating women, complementary feeding (children aged 6–23 mo)]	Studies focusing specifically on ready-to-use foods or food mixtures. Studies reporting specific food menus (e.g., for cancer patients or AIDS patients)	FBDGs provide advice on foods, food groups, and dietary patterns to ensure the intake of necessary nutrients for health and disease prevention. Population-specific FBDGs form the foundation for public health policies related to foods, nutrition, health, and agriculture, promoting healthy eating habits and lifestyles [5]. FBDGs are typically designed for all healthy individuals aged 2 y and above. However, many countries have also developed distinct guidelines for children under 2 y and specific population groups with unique nutritional needs, such as pregnant and lactating women and elderly people [40].
Study design	Analytical studies reporting the results of quantitative analyzes using either primary or secondary data sets in the form of peer-reviewed journal articles, conference proceedings, and university dissertations or theses	Descriptive studies, literature reviews (systematic/scoping), guidelines, commentaries, and other gray literature	This review aims to document the emerging strand of work using mathematical approaches in diet modeling. Documents that did not provide sufficient details for further discussion were not considered.
Language	English	Other languages	Translation of documents requires additional effort and resources, and several recent studies are available in the English language.
Timeline	January 2000–May 2024	Before January 2000 and after May 2024	Since the 1998 publication of “Preparation and Use of Food-Based Dietary Guidelines,” resulting from a joint WHO/FAO consultation, there has been growing international attention on the development of dietary recommendations, accompanied by a significant increase in research dedicated to this area.

Abbreviations: FBDG, food-based dietary guidelines; SSA, sub-Saharan Africa.

1 Countries in the SSA region includes Angola, Benin, Botswana, Burkina Faso, Burundi, Cape Verde, Cameroon, Central African Republic, Chad, Comoros, Democratic Republic of Congo, Republic of Congo, Cote d’Ivoire, Equatorial Guinea, Eritrea, Eswatini, Ethiopia, Gabon, Gambia, Ghana, Guinea, Guinea Bissau, Kenya, Lesotho, Liberia, Madagascar, Malawi, Mali, Mauritania, Mauritius, Mozambique, Namibia, Niger, Nigeria, Rwanda, Sao Tome and Principe, Senegal, Seychelles, Sierra Leone, Somalis, South Africa, South Sudan, Sudan, Tanzania, Togo, Uganda, Zambia, and Zimbabwe.

We imported all documents identified based on the search strategy into a Microsoft Excel spreadsheet, which we then used in screening and extracting data from the articles selected for the review. After removing duplicates, both authors (SS and MGDA) independently screened all extracted records according to the eligibility criteria. To screen the records, we followed an iterative process of screening the title and abstract, followed by the retrieval of full-text articles. Any disagreement between the authors on screening and data extraction was resolved through discussion and consensus. We collaboratively developed a data-charting form to identify the variables for extraction. The charted data underwent continuous updates in an iterative process, with discussions and consensus among the reviewers. To systematically document and summarize the evidence on the use of LP in providing dietary recommendations in SSA, we extracted the following items from each document into a preformatted spreadsheet by 1 author, while a second author randomly verified the subset of the extractions [30]: type of document; authors; year of publication; title of the article; country and location within the country; study population; aim and objectives; study design; methodological approach, including the use of software; and outcome (diet or dietary recommendations). In accordance with the standard practice of scoping reviews, we made no appraisal regarding the quality of the reviewed articles [41].

## Results

**FIGURE 1:**
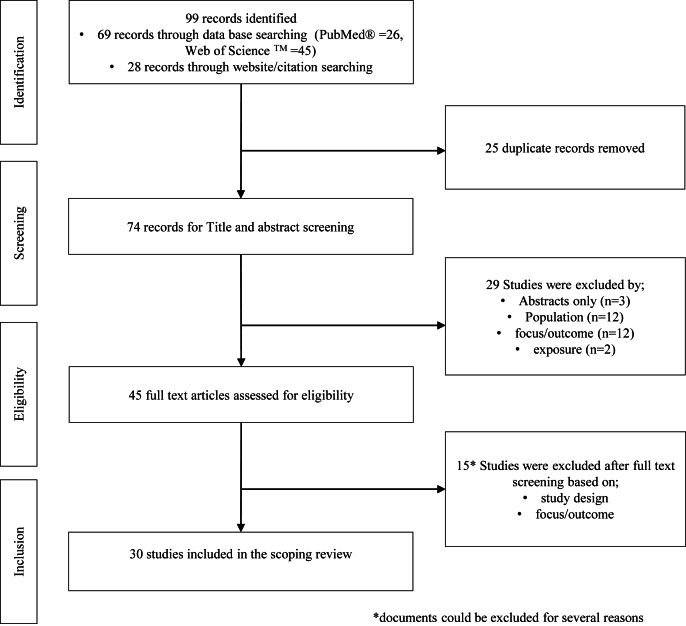
PRISMA flowchart for the selection of studies for the scoping review of use of mathematical programming in providing food based dietary recommendations in sub-Saharan Africa, 2000–2024.

Figure 1 presents the PRISMA flow diagram of the article selection process. The initial search of the 2 databases and citation search yielded 99 records. The number of articles identified has increased gradually, with the largest being 14, recorded in 2023. Following the removal of duplicates (*n* = 25), we retrieved and screened 74 records for titles and abstracts, of which 29 were removed for not meeting the inclusion criteria. We assessed the remaining 45 articles for eligibility and excluded 15 of them for the following reasons: 2 articles developed FBRs for very specific groups and 13 did not directly formulate FBRs using mathematical modeling but applied LP to other objectives, such as defining fortification strategies and developing food products and recipe formulas. Finally, 30 peer-reviewed scientific studies were included in the scoping review.

### Characteristics of the studies included

#### Area

The review encompassed 30 articles from 12 of the 48 countries in SSA, with studies distributed across Eastern, Western, and Southern Africa (subregion classification is based on the UN geoscheme for Africa; available on https://unstats.un.org/unsd/methodology/m49/). The highest concentration of studies was found in Eastern Africa, accounting for 22 articles. Within this region, Kenya contributed the most (*n* = 9), followed by Ethiopia (*n* = 4), Uganda (*n* = 3)—2 of which used the same data set for different objectives—Malawi (*n* = 2), Tanzania (*n* = 2), Zambia (*n* = 1), and Mozambique (*n* = 1). These studies primarily modeled optimal diets, addressing nutrient adequacy, affordability, and cultural relevance within local contexts. In Western Africa, 7 studies were identified. These included contributions from Ghana (*n* = 4), Benin (*n* = 1), Burkina Faso (*n* = 1), and Niger (*n* = 1). Meanwhile, Southern Africa was represented by a single study from South Africa.

**FIGURE 2:**
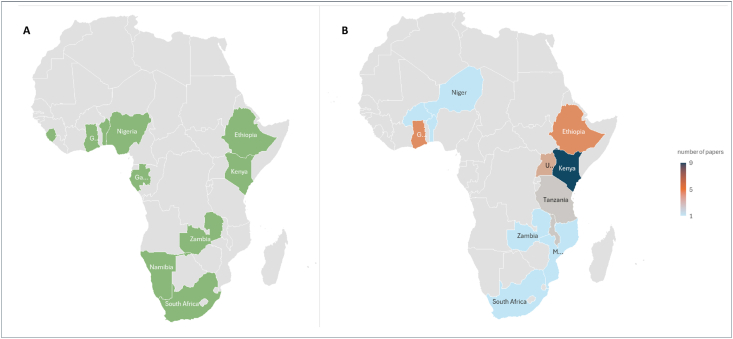
(A) Countries that have national food based dietary guidelines in the sub-Saharan Africa region. (B) The geographical distribution of 30 articles included in the review across the sub-Saharan Africa region.

Most of the research was conducted in rural settings; however, a few exceptions included studies in urban Burkina Faso and urban slums in Ethiopia. Among the 12 countries included, it is noteworthy that 6 nations—Benin, Ethiopia, Gabon, Ghana, Kenya, Zambia, and South Africa—have national FBDGs, as referenced in the FAO Dietary Guideline Repository [40] (Figure 2). Furthermore, it appears that these countries, which possess national FBDGs, also exhibit a higher volume of research related to diet modeling and related themes, as indicated by the significant number of published articles emerging from these nations.

#### Purposes

Table 2 [2,10,12,17,20,[27], [28], [29],[42], [43], [44], [45], [46], [47], [48], [49], [50], [51], [52], [53], [54], [55], [56], [57], [58], [59], [60], [61], [62], [63]] summarizes characteristics of the 30 studies included in the detailed review. The primary aim of these studies was to use LP to design nutritionally adequate and cost-effective diets or food baskets and to formulate FBRs with minimal deviation from prevailing diets. Notably, unlike dietary modeling in high-resource settings, where the focus is often on chronic disease prevention, these studies primarily emphasize achieving nutritional adequacy and affordability. LP analysis also helps identify “problem nutrients” (those that are difficult to meet using local foods within existing dietary patterns) and suitable food sources to improve nutrient intake for supporting better health outcomes. Approximately 80% of these studies focused on developing FBRs and the remainder targeted dietary optimization for specific deficient or problematic nutrients. The specific focus of the reviewed studies included formulating realistic and affordable FBRs by optimizing the current dietary patterns using locally available foods to meet nutritional needs and gaps (*n* = 14); assessing the feasibility of introducing wild food (*n* = 1), novel foods (i.e., house crickets; *n* = 1), locally available nutrient-rich foods (i.e., yellow cassava and fermented milk; *n* = 2), and fortified products (*n* = 3) to address nutrient deficiencies in the current diet; developing nutritionally and culturally optimized and cost-minimized food baskets for a typical household or an adult (*n* = 4); describing the use of LP as a method of designing population-specific FBDGs and formulating nutrient-adequate diets (*n* = 2). There were studies that focused on modeling diets that fulfill the diet goals recommended in their country-specific FBDGs (e.g., Benin and Ghana; *n* = 2), which also addressed the dietary recommendations for preventing chronic health diseases, and 1 study analyzed the development of FBRs, while identifying key contextual factors impacting their implementation.

**TABLE 2:** Summary of the studies included in the scoping review on use of diet modeling in developing food-based recommendations in sub-Saharan Africa (sorted by publication year).

Reference	Study setting	Study aim	Outcome/finding	Comment related to modeled diet
[2]	Rural Malawi	To describe the use of LP as a method of formulating a nutrient-adequate diet	Season-specific FBRs for children aged 3–6 y using locally available foods	During the harvest season, nutritional needs can be met with minor deviations from the local diet. In the nonharvest season, limited availability of riboflavin-rich and zinc-rich foods and high phytate concentrations made it challenging to meet those needs.
[12]	Rural Malawi	To develop an approach using LP for designing population-specific FBDGs	Season-specific FBRs for children aged 3–6 y using locally available foods	A daily diet comprising maize flour, small-dried fish, leaf relish, and 2–3 snacks. During food shortages, including mangoes and legume relish; during plentiful times, pumpkins improve nutrient intake.
[42]	Ghana	To determine the least-cost food basket that satisfies recommended nutritional requirements using LP	A nutritional and economically optimized daily food basket of an average Ghanaian (22-y-old males)	A food basket comprising sorghum, yam, cassava, coconut, and milk can meet nutritional requirements and save 80% of daily earnings.
[43]	Kenya	To determine the role of wild food biodiversity in a least-cost nutritious diet using LP	Locally adapted, cost-optimized nutritious diet for children aged 6–23 mo, women, lactating women, and pregnant women	Modeled diets excluding wild species were deficient in iron, vitamin B-6, and calcium during the dry season. Including wild foods reduced costs and enabled women and children to meet their iron requirements.
[10]	Mozambique	To assess the role of local foods in diet diversification using LP	Low-cost, fully nutritious food basket for a family of 6	Dietary diversification strategies using local, low-cost, nutrient-dense foods can meet all micronutrient recommendations.
[17]	Southern Benin	To establish the recommended daily servings and portion sizes for the Benin food guide	The number of servings per food group and the portion size for 8 age–sex groups	Local diets could be optimized to meet only 70% of the limiting micronutrient (iron, calcium, folate, and zinc).
[44]	Rural Kenya	To identify realistic FBRs using LP	FBRs for children aged 6–23 mo	Meeting iron, zinc, and calcium needs with local foods is challenging. Breastfed children should have cow or goat milk (in porridges), fortified cereals, GLVs, legumes, and meat, fish, or eggs to ensure dietary adequacy of ≥7–9 of 12 nutrients.
[45]	Rural Kenya	To derive FBRs and identify contextual factors affecting the implementation of FBRs using LP	FBRs for children aged 6–23 mo	Iron, zinc, and calcium are the problem nutrients. To meet nutritional needs, it is important to either introduce new food sources or significantly improve access to existing ones.
[46]	Northern Kenya	To formulate age-specific and context-specific CFRs using LP	Age-specific and context-specific CFRs for children aged 6–23 mo	Age-specific CFR could achieve adequacy for 7–9 of the 11 modeled micronutrients. Modeled diets include animal milk, potato or green banana, grains and grain products, beans, vitamin A–fortified fats or oils, and fruits.
[29]	Rural Tanzania	To formulate realistic and affordable FBRs that meet DRIs using LP	Optimal diets for children aged 6–23 mo	Optimized diet can achieve DRIs of 20 nutrients and contains locally sourced wholegrain cereals, Irish potatoes, pulses and seeds, fish and poultry meat, fruits, and vegetables.
[47]	Urban Burkina Faso	To formulate nutritious and affordable diets using local foods using LP	Lowest-cost optimal diets for women of reproductive age	The diet can be optimized to meet micronutrient needs by including rarely consumed nutrient-dense foods such as nuts, seeds, DGLVs, certain fruits, and liver into daily diets.
[48]	Ghana	To formulate nutritionally optimized, culturally acceptable, and cost-minimized diet using LP	Optimized diet for low-income Ghanaian families	Optimization suggested reducing roots, tubers, and fruits, whereas increasing cereals, vegetables, and oil-bearing crops compared with the food balance sheet.
[49]	Kenya	To formulate and test FBRs using LP	FBRs with zinc-fortified water for children aged 4–6 y	The modeled diet covered 87% of the zinc RNI and included whole grains, milk, nuts, vitamin A–rich and vitamin C–rich vegetables, starchy plants, and small whole fish with bones.
[50]	Kenya	To assess the potential effect of introducing a yellow cassava-based school lunch combined with additional FBRs using LP	FBRs with yellow cassava and other alternative interventions for children aged 7–9 y	The modeled diet includes oil, dairy products, grain products, legumes, nuts or seeds, small, dried fish, yellow cassava, and vegetables. Optimized diet could not ensure an adequate intake of fat, riboflavin, folate, and vitamin A.
[51]	Tanzania	To formulate a nutritious, realistic, and affordable diet using LP	Optimal dietary pattern formulated from locally available foods for rural pregnant and lactating women	Optimized diet includes 12 food groups: cereals; roots, tubers, and plantains; pulses, nuts, and seeds; vegetables; fruits; meat, poultry, and insects; eggs; fish and sea food; milk and dairy products; milk and dairy products; fats and oils; sweets; and spices; condiments, and beverages, as represented by local food items.
[52]	Kenya	To develop FBDGs using LP	FBRs for rural Kenyan women	Optimized diet includes fruits, vegetables, dairy products, added fats, grains and grain products, legumes nuts and seeds, snacks, Sukuma wiki, cow milk, fat mallo, maize flour, and dried red beans.
[53]	Ethiopia	To identify nutritionally an adequate and affordable FB using LP	Cost-minimized, nutritionally adequate, culturally adopted FB that can be served as a basis for the development of FBDGs for low-income population	The optimized diet composed of ∼60% cereals, 20% roots and tubers, 10% legumes, and 10% fruits and vegetables by weight, with only a small share from ASFs.
[54]	Niger	To identify nutrient gaps and develop FBRs	FBRs with additional supplementation strategies to improve dietary micronutrient adequacy for pregnant and lactating women	It is challenging to model a diet to meet dietary allowances for all micronutrients, except for vitamin B-6, iron, and zinc. FBRs include daily consumption of DGLVs, fermented milk, millet, pulses, and vitamin A–fortified oil, and 1 additional meal per day.
[55]	Ethiopia	To identify dietary strategies to improve nutrient adequacy using LP	A set of regional FBRs with some alternative interventions for children (6–23 mo)	Combination of regional FBRs for local complementary foods with micronutrient powder supplementation can close the identified nutrient gaps.
[28]	Ghana	To develop FBDGs that cover nutrient adequacy within the constraints of local current dietary patterns	A set of FBDGs for infants and young children (6–23 mo)	FBRs with breast milk, vegetables, dairy, whole grains, fruits, fish, nuts or seeds, beans, and additional extra recommendations for grain legumes (cowpea and soybean) can improve the adequacy of calcium, iron, niacin, and zinc.
[56]	Kenya	To model optimized lowest-cost diets for women and children in rural Kenya	A locally adapted cost-optimized nutritious diet for women and children’s diet	Locally adapted cost-optimized nutritious diets modeled with wild vegetables increase micronutrient intake and optimized cost.
[57]	Zambia	To formulate FBRs using LP to improve dietary nutrient intake	FBRs combining fermented milk products with local food patterns for children aged 1–5 y	FBRs combining local food pattern with fermented milk products improve nutrient intake, except for iron and zinc.
[27]	Ethiopia/urban slums	To rationally combine local foods to provide basic nutritional needs whereas limiting the use of commercial foods using LP	Age-specific (6–23 mo) FBRs	An optimized diet, including local foods slightly complemented with commercial foods, can achieve optimal dietary intakes of essential nutrients.
[58]	South Africa	To determine whether the nutrient requirement of infants can be achieved with a food-based approach using LP	A fully nutritionally optimized diet for infants (6–11 mo) cannot be achieved either with the current food pattern or with no pattern	The best modeled diet would provide nutrient adequacy of energy, protein and 8 of the 11 micronutrients, if breastfeeding on demand continues during the complementary feeding phase.
[59]	Rural Uganda	To provide realistic FBRs for improving nutrition using LP	An optimized diet for breastfeeding 12- to 23-mo-old children	FBRs include food choices of cow milk, legumes, green leafy vegetables, sweet potatoes, and fruits. Modeled diet can achieve dietary adequacy for 8 of the 12 nutrients.
[60]	Five urban and rural regions in Ethiopia	To develop a healthy diet that is closely related to the current diet and takes the fasting period into account using linear goal programming	Optimized modeled diet for women of reproductive age that provides an adequate intake of most of the targeted micronutrients	Compared with the current diet, the optimized modeled diets include higher intake amounts from food groups, milk and dairy products, nuts and seeds, and fruits. The cost of the optimized diet is twice that of the current diet.
[20]	Ghana	To formulate a healthy diet that best meets the dietary goals established for the FBDGs in Ghana using LP	An optimized diet for the adolescents and adult populations	The optimized diet includes ASFs, discretionary choices, fruit, vegetables, healthy fats and oil, legumes, nuts, seeds, and staples. A lower ASF in the modeled diet is known to have lesser impact on the environment.
[61]	Kenya	To develop FBRs using LP	FBRs based on locally available foods for children aged 2–3 y	FBRs include house crickets as a source of zinc and nutrient-dense foods, including oil, mango, sour cow milk, butternut, and fruits.
[62]	Urban and rural Uganda	To develop FBRs using LP to improve calcium intake	Optimized diets containing fortified products with calcium-rich local foods for women, adolescent girls, and young children	Optimized diets containing fortified products, with calcium-rich local foods.
[63]		To develop FBRs using locally available foods to improve the calcium intake	FBRs with calcium optimized diets for infants, women, young children, and adolescent girls	FBRs include GLVs and milk, and species of small fish, lime-treated maize products, sesame seeds, and bean varieties as best food sources of calcium.

Abbreviations: ASF, animal-source food; CFR, complementary feeding recommendations; DGLV, dark green leafy vegetable; DRI, dietary reference intake; FB, food basket; FBDG, food-based dietary guidelines; FBR, food-based recommendation; GLV, green leafy vegetable; LP, linear programming; RNI, recommended nutrient intake.

#### Age groups and subgroups

**FIGURE 3:**
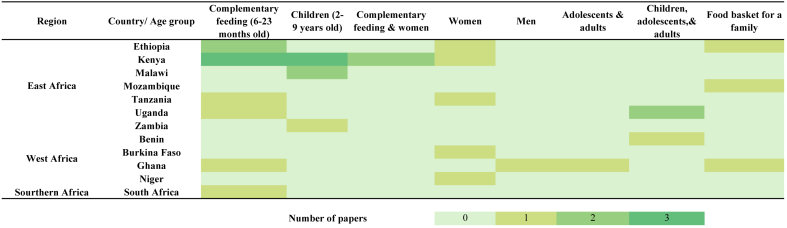
Age group considered developing food-based recommendations in the studies included in the detailed review.

Figure 3 summarizes the age groups covers in the included studies. According to Table 2 and Figure 3, all studies used mathematical modeling to optimize dietary outcomes across various age groups, including infants (6–23 months), young children, adolescents, and adults in SSA. Notably, formulated FBRs and optimized diets often focus on specific and vulnerable demographic groups with a limited geographic scope. These target populations reflect regional priorities for addressing undernutrition in vulnerable groups, such as children and women of reproductive age—for instance, infants and children aged 6–23 mo (*n* = 10); children aged 3–9 y (*n* = 6); women, pregnant women, lactating women, and women of reproductive age (*n* = 5); and different demographic groups with specific nutritional needs (i.e., children, women, and adolescent girls; *n* = 4). Some studies explored broader family diets, such as food baskets for a household (*n* = 3), and some focused on formulating an optimized diet for an average adult (*n* = 2).

#### Mathematical approaches

Although we primarily focused on reviewing the utilization of mathematical approaches in diet modeling and the development of FBRs, it is noteworthy that 24 of the 30 articles used LP analysis, whereas 6 used linear goal programming [2,12,29,54,60], an extension of LP. Table 3 [2,10,12,17,20,[27], [28], [29],[42], [43], [44], [45], [46], [47], [48], [49], [50], [51], [52], [53], [54], [55],[57], [58], [59], [60], [61], [62], [63]] presents a summary of the LP analyses conducted in the included studies.

**TABLE 3:** Summary of details retrieved from articles that used linear programming for modeling optimized diet and proposing food-based recommendations.

Country	Reference	Diet of	Dietary data	Focus	Program/software	Optimization (objective function/s)	Nutritional constraints	Other constraints^1^
East Africa								
Ethiopia	[27]	Children aged 6–23 mo	Cross-sectional	Nutritionally optimized diet using local food	ProPAN, NutriSurvey	Nutritionally optimized CF diet	RNIs of energy, fat, protein, carbohydrates, iron, zinc, calcium, and vitamins A and C	Food use limits (minimum and maximum serving size/d), cost
	[55]	Children aged 6–23 mo	Cross-sectional	Formulating feasible FBRs for children in 3 age groups in 4 regions in Ethiopia	Optifood	To minimize deviations below the RNI and deviations from observed food pattern	Energy requirement of the target group and RNIs of 13 nutrients^2^	Food groups, average serving size, and frequency of each food item consumption
	[60]	Women of reproductive age	Cross-sectional (*n* = 494)	Healthy diet resembles the current diet and considering fasting periods	FICO Xpress 8.10	Minimizing the largest unwanted nutrient intake deviation	Energy intake, EAR, and RDA of protein, iron, zinc, calcium, and vitamins A, B-6, and B-12, thiamine, riboflavin, niacin, folate	Number of food items consumed in a food group and subgroups
	[53]	A family of 5 members	Food balance sheet (FAO)	Nutritionally adequate, affordable, and acceptable FB for a family of 4	MS Excel OpenSolver	To minimize the cost of the optimized FB and the RD from the standard EER	REI, RNIs protein, fat, SFAs, PUFAs, ω-3 PUFAs, ω-6PUFAs, TFAs, niacin, folate cholesterol, carbohydrate, fiber, total sugars, sodium, potassium, calcium, magnesium, iron, zinc, selenium, iodine, vitamin A (RAE), thiamine, riboflavin, vitamins B-6, B-12, C, and E	Cost, acceptability
Kenya	[45]	Children aged 6–23 mo	Cross-sectional (*n* = 335)	Formulating FBRs for children and identifying contextual factors affecting their implementation	Optifood	To minimize cost and optimize nutrient intake adequacy	Energy requirement of the target group and RNIs of 13 nutrients	Median portion size per serving, minimum and maximum number of servings of the selected foods/week
	[46]	Children aged 6–23 mo	Cross-sectional (*n* = 882)	Formulating age-specific and context-specific complementary feeding recommendations	Optifood	To optimize nutrient adequacy	Energy requirement of the target group and RNIs of 13 nutrients	Median portion size per serving, minimum and maximum number of servings/week
	[44]	Children aged 6–23 mo	Cross-sectional/24 h (*n* = 401)	Locally appropriate FBRs that are low cost and improve nutrient adequacy	Optifood	To minimize cost and optimize nutrient intake adequacy	Energy requirement of the target group and RNIs of 13 nutrients	Minimum and maximum serving from food group and weight (g) of each food item/week
	[61]	Children aged 2–3 y	Cross-sectional (*n* = 47)	FBRs based on local available foods with house crickets as a zinc source	Optifood	Minimizing the deviation from the average food pattern	Energy requirement of the target group and RNIs of 13 nutrients	Minimum and maximum number of servings/week
	[49]	Children aged 4–6 y	Cross-sectional (*n* = 112)	Developing and testing FBRs that included zinc-fortified water	Optifood	To minimize the deviation from average food pattern	Energy requirement of the target group and RNIs of 13 nutrients	Minimum and maximum number of servings/week
	[50]	Children aged 7–9 y	Cross-sectional (*n* = 150)	FBR based on the potential inclusion of yellow cassava into diet	Optifood	To minimize the deviation from current food pattern	Energy requirement of the target group and RNIs of 13 nutrients	Median serving sizes, food cost, and food (sub)group frequency distributions
	[56]	Children aged 6–23 mo and women aged 15–49 y	Cross-sectional (*n* = 180)	Lowest cost, nutritionally optimized diet	Cost of diet	To minimize the cost of the diet	RNIs energy, protein, fat, vitamins A, C, B-1, B-2, niacin, B-6, folic acid, and B-12, calcium, iron, zinc, copper	Minimum and maximum food intake/week
	[43]	Children aged 6–23 mo, women, lactating and pregnant women	Food inventory compiled in a market survey	To screen wild foods for their potential to reduce diet costs and meet nutritional requirement	Cost of diet	To minimize the cost	RNIs energy, protein, fat, vitamins A, C, B-1, B-2, niacin, B-6, folic acid, and B-12, calcium, iron, zinc, copper	Minimum and maximum number of times a food can be included in the diet, cultural acceptability
	[52]	Rural women aged 15–49 y	Cross-sectional (*n* = 210)	Formulating FBRs to improve dietary quality	Optifood	To optimize nutrient adequacy	Energy requirement of the target group and RNIs of 13 nutrients	Average portion size, frequency of food consumption/week
Malawi	[12]	Children aged 3–6 y	3-d weighted food records (*n* = 79)	Design, test, and refine population-specific FBDGs	MS Excel Solver	To minimize the difference between the observed diet and quantity of nutrients in the modeled diet	Energy, RNIs (protein, calcium, iron, folate, vitamins B-12, C, A, thiamine, riboflavin, and niacin, zinc, copper) and molar ratio of phytate to zinc	Acceptability (realistic diet types, 3-d portion size, number of 1-d portions of main meal dishes and snacks)
	[2]	Children aged 3–6 y	Cross-sectional/3-d weighted food records (*n* = 65)	Nutrient-adequate diets of optimal nutrient density	MS Excel Solver	To minimize the total energy content of the optimized diet	RNIs (protein, calcium, iron, folate, vitamins B-12, C, A, thiamine, riboflavin, and niacin, zinc, copper) and molar ratio of phytate to zinc	Lower and upper limits of energy intake from different food groups, daily portion size of each food
Mozambique	[10]	A family of 6 members	Commonly consumed foods	Nutritionally optimized low-cost diet with local foods	MS Excel Solver	To minimize the cost of food basket	DRIs energy, protein, fat, calcium, magnesium, zinc, iron, vitamins B-1, B-2, B-6, B-12, C, E, and A, niacin, pantothenic acid, folate	
Tanzania	[29]	Children aged 6–23 mo	Cross-sectional (*n* = 400)	Realistic and affordable diets that achieve DRIs for 22 selected nutrients	LiPS version 1.9.4	To minimize the deviations from the population’s food subgroup patterns for 11 food subgroups	EER, DRIs of protein, calcium, iron , zinc, copper, magnesium, manganese, phosphorus, potassium, sodium, vitamins A, B-1, B-2, B-6, B-12, C, D, E, folate, niacin, and pantothenic acid	Minimum and maximum food use limits and cost
	[51]	Pregnant and lactating women	Cross-sectional (*n* = 150)	Formulating a nutritious, realistic and affordable diet using locally available foods	Linear Program Solver	To minimize the deviation from the population’s food consumption pattern	RNIs of energy, protein and amino acids, vitamins A, D, E, K, and C, calcium, niacin, thiamine, iodine, riboflavin, vitamins B-6 and B-12, biotin, pantothenic acid, selenium, zinc, iron, magnesium, folate, and folic acid	Minimum and maximum quantity of each food item, food cost
Uganda	[59]	Children aged 12–23 mo	Cross-sectional (*n* = 114)	Generate and test FBRs to identify the best food sources from local foods	Optifood	Nutritionally optimized complementary feeding diet	Energy requirement of the target group and RNIs of 13 nutrients	Minimum and maximum servings/food/week; equality constraint on the diet’s energy content
	[62]	Women, adolescent girls, and young children	National panel survey/household consumption and expenditure survey	FBRs with fortified products to fill the calcium intake gap of the population	Optifood	Maximize the calcium intake	Energy requirement of the target group and RNIs of 13 nutrients	Minimum and maximum number of servings/week
	[63]			FBRs with local foods to meet the calcium population reference intake	Optifood	Maximize the calcium intake	Energy requirement of the target group and RNIs of 13 nutrients	Minimum and maximum number of servings/week
Zambia	[57]	Children aged 1–5 y	Cross-sectional (*n* = 221)	FBRs from locally available cereal and milk based fermented foods	Optifood	A realistic diet that satisfies the model parameters	Energy requirement of the target group and RNIs of 13 nutrients	Minimum and maximum servings/food/week
Southern Africa								
South Africa	[58]	Children aged 6–11 mo	Cross-sectional (*n* = 134)	A food-based approach to ensure nutrient adequacy	Optifood	Nutritionally optimized CF diet	Energy requirement of the target group and RNIs of 13 nutrients	Min and max servings/food/week
Western Africa								
Benin	[17]	Children, Adolescents and adults	Cross-sectional/24 h (*n* = 541)	Daily number of servings per food group and the portion sizes of common foods to be recommended for the Benin food guide	MS Excel Solver	To minimize the difference from observed diet and quantity of a nutrient in the modeled diet	RNIs energy, macronutrients, 12 micronutrients, and dietary guidelines for the prevention of chronic diseases	Maximum amount per food category and food item
Burkina Faso	[47]	Women of reproductive age	Cross-sectional (*n* = 182)	Feasibility of achieving low-cost nutritious diet using local foods alone and local foods and supplements	MS Excel Solver	To minimize the cost of the diet	EER, AMDR, RNIs of vitamins A, C, B-6, B-12, folate, thiamine, and riboflavin, calcium, iron and zinc	≥30% of calories from starchy staples, maximum quantities for food items, and food groups
Ghana	[28]	Children aged 6–23 mo	Cross-sectional	FBDGs with legumes to supplement protein and micronutrient	Optifood	To minimize the deviation of the current diet	Energy requirement of the target group and RNIs of 13 nutrients	Frequency of servings
	[42]	22-y-old man	Food balance sheet	A cost-minimized diet that meets the nutritional requirements	Not mentioned	To minimize the food expenditure	RDIs of energy, protein, carbs, vitamins A and C, iron and calcium	Maximum amount of daily food consumption
	[20]	Adolescents and adult population	National food survey	Healthy diet that meets the FBDGs for Ghana	DietSolve	Minimizing the deviation from the energy target	Percentage energy from carbohydrates, protein, fat, and RNIs of calcium, iron and zinc	Minimum and maximum servings/day for each food group
	[48]	Rural and urban HH		Nutritionally optimized FB for a low-income family of four	MS Excel OpenSolver	To minimize total cost of FB	REI, RNIs of protein, fat, SFAs, PUFAs, ω-3 PUFAs, ω-6 PUFAs, TFAs, niacin, fiber, cholesterol, carbohydrate, total sugars, sodium, potassium, calcium, magnesium, iron, zinc, selenium, iodine, vitamin A (RAE), thiamine, folate, riboflavin, and vitamins B-6, B-12, C, and E	Minimum deviations from the prevailing food supply
Niger	[54]	Pregnant and lactating women	Cross-sectional (*n* = 202)	FBRS to improve micronutrient adequacy	Optifood	To optimize nutrient adequacy	Energy requirement of the target group and RNIs of 13 nutrients	Median serving per week for each food group

Abbreviations: AMDR, accepted macronutrient distribution range; CF, complementary feeding; DRI, dietary reference intake; EAR, estimated average requirement; EER, estimated energy requirement; FB, food basket; FBDG, food-based dietary guideline; FBR, food-based recommendation; RAE, retinol activity equivalent; RD, relative deviation; RDA, recommended daily allowance; RDI, recommended daily intake; REI, recommended energy intake; RNI, recommended nutrient intake; SFA, saturated fatty acid; TFA, trans fatty acid.

1 Palatability/social and cultural acceptability/environmental constraint.

2 The in-built nutritional constraints of the Optifood software include energy requirements of the target group (27 demographic groups ranging from infants to the elderly), RNIs of 13 nutrients; fat; protein; calcium; iron; zinc; and vitamins A, C, B-6, and B-12, thiamine, riboflavin, niacin, and folate drawn from WHO/FAO/UNU guidelines.

In LP analysis, the objective function indicates the contribution of the decision variables to the value of the function to be optimized. For LP diet optimization, the objective is to identify a combination of foods that satisfies nutritional requirements at a minimal cost or to find a combination of foods, which is minimally deviated from the current habitual diet. In some cases, it also takes into account factors such as cultural and social acceptability and environmental sustainability, ensuring that all defined constraints for effective diet optimization are incorporated [24,26].

The objective functions across studies included minimizing deviations between observed diet and optimized diet to align with the average food pattern (*n* = 10), minimizing dietary energy content (*n* = 2), minimizing diet costs or food expenditure (*n* = 9), optimizing nutrient adequacy (*n* = 6), and maximizing the intake of “problem nutrients,” the nutrients that are most difficult to achieve the recommended adequacy (*n* = 2).

Decision variables, which are factors controlled by the decision maker and must be determined to solve an LP problem. In all studies, the quantity of the food item, food subgroup, or food group, expressed in grams, was used as the decision variable. Constraints are typically imposed on the decision variables. Nutritional constraints such as recommended nutrient intake and estimated energy requirements are consistently applied across studies. Diet optimization problems used in the studies were subjected to several constraints, either to optimize the energy requirement of the target demographic group and recommended nutrient intakes of macronutrients and micronutrients (*n* = 30), constraints on economic issues (food cost, *n* = 4), or acceptability (*n* = 30). Studies often impose additional constraints on acceptability, palatability, and local dietary habits that ensure a minimal deviation from the current diets, which are crucial for the successful implementation of dietary guidelines. For example, Levesque et al. [17] accounted for local eating habits in Benin, and Termote et al. [43] considered cultural acceptability when evaluating wild foods as potential components of an optimized diet. No study used constraints related to environmental issues or sustainable diets (e.g., greenhouse gas emissions, energy use, land use, or exposure to contaminants).

The dietary data used in these studies typically originates from cross-sectional surveys (*n* = 21), which collected 24-h, 3-d, and 7-d diet recalls; national food surveys or market surveys (*n* = 7); and nationally representative food balance sheets (*n* = 2; i.e., FAO food balance sheet).

#### Computer programs

LP was central to these studies, with various software tools used, including Optifood (*n* = 15), MS Excel Solver (*n* = 7), Cost of Diet (*n* = 2), Linear Program Solver (*n* = 2), FICO Xpress (*n* = 1), ProPAN (*n* = 1), and DietSolve (*n* = 1).

##### Optifood

Half the studies used the Optifood software, an advanced computerized LP analysis tool. It enables public health professionals to identify the nutrients derived from local diets and formulate and test population-specific FBRs to meet their nutritional needs [64]. The effectiveness of this approach is in mathematical optimization, which considers dietary patterns and estimated energy requirements and 13 essential nutrients, which are too computationally intensive to perform manually [65].

The Optifood software performs LP analyses using 4 analytical modules—module I: check diets; module II: identify draft recommendations; module III: test FBR; and module IV: cost analyses (optional module) [66]. Optifood has been tested or used in 10 countries across Asia, Africa, and Latin America and has proven useful in developing nutritional improvement strategies [66]. However, it is not yet available for public download.

##### MS Excel solver

Microsoft Excel with its built-in solver function is one of the LP tools widely accessible on personal computers and effective for diet optimization. This program accommodates a wide range of constraints and objective functions, making it a versatile research tool. The process begins by creating a tabular food database and then activating the solver function through the Tools menu [11]. Although Excel’s solver function is accessible and versatile, it requires manual setup and is less suited for fieldwork compared with specialized tools [11].

##### Other software

Process for the Promotion of Child Feeding (ProPAN) uses both qualitative and quantitative methods to evaluate breastfeeding and complementary feeding practices at the local level, facilitating the development of culturally appropriate and acceptable feeding recommendations [67]. ProPAN can analyze anthropometric data (module I) and the energy and nutrient profiles of recipes (module II), as well as determine food consumption frequency, average food serving sizes, and the number of servings of foods provided from selected food groups and subgroups [66]. Other diet modeling tools using LP analyses include NutriSurvey, Cost of Diet [59], and DietSolve [20]. These tools differ from Optifood in their scope (e.g., the number and types of LP models analyzed) and the nature of the modeled diets.

### Type of FBRs or diets

Most of these studies (*n* = 14) have highlighted the importance of using food-based approaches to fill nutrient gaps by optimizing the current diet with locally available nutrient-dense food groups and food items, such as green leafy vegetables, milk and dairy products, small-dried fish, fruits, and legumes. In contrast, some studies (*n* = 7) have stated that modeled diets based solely on locally available foods cannot meet the nutritional adequacy of micronutrients such as Zn, Fe, Ca, and vitamin B-6. Slightly complementing the current diet with rarely consumed foods (i.e., wild foods, novel foods, and fermented milk), food supplements, or fortified foods can help achieve dietary adequacy (*n* = 7). Agricultural seasons significantly affect food availability; thus, season-specific diets must be formulated to meet nutritional requirements (*n* = 2). Several studies highlight seasonal variations, fasting practices, or urban–rural differences, reflecting sociocultural and environmental influences on diet design.

TABLE 4, TABLE 5 [2,10,12,17,20,[27], [28], [29],[42], [43], [44], [45], [46], [47], [48], [49], [50], [51], [52], [53], [54], [55], [56], [57], [58], [59], [60], [61], [62]] present a comprehensive summary of locally appropriate FBRs developed through LP for diverse population groups across multiple countries in SSA. The FBRs are expressed in diverse formats, including daily intake (grams per day), number of servings per day, and servings per week. These recommendations are derived from varying levels of dietary classification-ranging from broad food groups (e.g., cereals, roots and tubers, legumes) to more specific subgroups (e.g., pulses, dark green leafy vegetables, added fats, and sugar-sweetened beverages), and in some cases, individual food items (e.g., fortified cereals, maize flour, sweet potato, cowpea leaves, and *mabisi*). Several studies include context-specific foods such as house crickets [61], wild edible plants [43], yellow cassava [50], and traditionally fermented dairy products like *mabisi* [57]. Although food preparation methods are generally not specified, a few studies note the state of the food—such as cooked [54] or served as relish [12]—when relevant to the recommended form of consumption.

**TABLE 4:** Summary of the final set of locally appropriate FBRs included in the study for young children, adolescents, adults, and families.

Country (study)	Age group (unit in food group recommendation)	Food groups/subgroups [(g/d), (servings/d), or (servings/wk)]												
		Cereals	Tubers/other starch/roots	Nuts and seeds	Fruits	Vegetables	Legumes	Meat	Fish	Egg	Milk/milk products	Fat/oils	Salt and sugar	Other
East Africa														
Malawi [2]	3–6 y old (g/d)	310	Roots: 217	—	77	194	101	92			—	—	—	—
Malawi [12]	3–6 y old (g/d)													
	Food plenty season	≥205	—	—	—	≥180 pumpkin once every 3 d	—	—	≥20	—	—	—	—	≥70 leaf relish twice every 3 d
	Food shortage season	≥230	—	—	≥120	—	≥30 once every 3 d	—	≥20	—	—	—	—	≥70 leaf relish once every 3 d
Mozambique [10]	A family of 6 members (g/d)	1551	—	311	—	328	1254	258	—	498	—	—	—	—
Kenya [61]	2–3 y (servings/wk)													
	FBR (no house cricket diet)	Whole grains and products: 24, finger millet flour: 7, yellow maize flour: 7		—	5	Cowpea leaves: 3, DGLV: 3	2	—	4	—	7	Added fats: 9	—	—
	FBR (with house cricket diet)	Whole grains and products: 24, finger millet flour: 7, yellow maize flour: 7		—	5	Cowpea leaves: 3, DGLV: 3	—	House crickets: 3	—	—	7	Added fats: 11	—	—
Kenya [49]	4–6 y old (servings/wk)	7	1	4	—	14 (vitamin A–rich food: 7, vitamin C–rich food: 7)	—	—	7	—	7	—	—	—
Kenya [50]	7–9 y old (servings/d)	2	0.7	legumes, nuts & seeds: 2		4	legumes, nuts & seeds: 2	2	1			added fats: 1		
Kenya [52]	Women (servings/wk)	16	Tubers: 23, roots: 2	5	2	18	—	—	—	—	5	5	—	—
Tanzania [51]	Pregnant women (g/d)	434.3	—	Legumes, nuts and seeds: 90.8	16	408	—	20.1	62.3	0	45	2.2	14.2	Spices, condiments, beverages: 1.1
	Lactating women (g/d)	306.1	599	Legumes, nuts and seeds: 92.3	8	633.4	—	25	111.5	0	0	2.7	0	Spices, condiments, beverages: 0.7
Ethiopia [60]	Women of reproductive age (g/d)													
	Nonfasting diet	568		20	195	129	Pulses: 88	22			396	17	SSB: 29	Beverages, spices, and other: 322
	Continuous fasting	611		20	202	165	Pulses: 92	0			0	15	SSB: 30	Beverages, spices, and other: 338
	Intermittent fasting	588		21	194	132	Pulses: 90	16			298	16	SSB: 28	Beverages, spices, and other: 337
Ethiopia [53]	Family of 5 (g/d)													
	Rural	2082	660.9	Legumes and nuts: 365.3	378		Legumes and nuts: 365.3	55.7		5.4	45.7	46	40	Spices, condiments: 26.1
	Urban	2022.7	502.8	Legumes and nuts: 319.9	332.1		Legumes and nuts: 319.9	28		4.8	126.8	40.5	24.4	Spices, condiments: 24.8
Zambia [57]	1–3 y old (servings/wk)	7	—	4	—	21; rape leaves: 7	Beans, lentils, and peas: 3	—	2	—	*Mabisi*: 7	—	—	—
	4–5 y old (servings/wk)	6	—	5	—	21; rape leaves: 7	Beans, lentils and peas: 3	—	—	—	*Mabisi*: 6	—	—	—
Uganda [62]	12–23 mo, 4–6 y, 10–14: y: old girls, NPNL women (servings/wk)													
	Central area: rural	—	—	—	—	GLV: 6	Beans: 7	—	7	—	4	—	—	—
	Central area: urban	—	Sweet potatoes: 7	—	—	GLV: 7	—	—	7	—	4	—	—	—
	Northern: rural	—	—	Sesame: 6	—	GLV: 7	—	—	7	—	—	—	—	—
	Northern: urban	—	—	Sesame: 6	—	GLV: 7	—	—	7	—	—	—	—	—
West Africa														
Ghana [42]	22: y: old males (g/d)	173	Tubers: 58, roots: 348	212 coconuts	—	—	—	—	—	—	747	—	—	—
Ghana [20]	Adults (g/d)	671.8		—	225.7	227.3	Legumes, pulses, and nuts: 197.6	ASF: 144.2	—	—	—	6.2	—	Discretionary choices: 41.5
Ghana [48]	A family of 4 (g/d)													
	Urban	1905	—	333	—	358	2	10	—	—	—	84	17	—
	Rural	638	Tubers: 726, roots: 297	—	171	637	14	—	—	—	6	17	—	—
	Rural with wild foods	—	Tubers: 900, roots: 318	—	—	125	4	—	—	—	—	144	15	Wild foods: 3737
Benin [17]	Different age groups (servings/week)													
	2–3 y old	2–3		—	1	2–3	1				1	—	—	—
	4–8 y old	2–4		—	1–3	3–5	1–2				1	—	—	—
	9–13 y old	3–4		—	2–3	4–5	1–2				1	—	—	—
	14–18: y: old girls	4–6		—	2–3	4–6	2–3				1–2	—	—	—
	14–18: y: old boys	5–7		—	2–3	5–6	2–3				2	—	—	—
	Women	3–5		—	2–3	4–6	2–3				1–2	—	—	—
	Men	4–6		—	2–3	4–6	2–3				1–2	—	—	—
	Lactating women	5–6		—	2–3	4–6	3				1–2	—	—	—
	Pregnant women	4–6		—	2–3	4–6	2–3				1–2	—	—	—
Burkina Faso [47]	NPNL women (g/d)													
	All local foods	363	32	44	56	40	83	4	0			27		
	Common local foods	338	0	44	360	294	104	58	6			12		
Niger [54]	Pregnant women (servings/wk)	Millet: 14	—	—		DGLV: 21	Cooked: 21	—	—	—	14	Vitamin A–fortified vegetable oil: 21	—	—
	Lactating women (servings/wk)	Millet: 14	—	—	—	DGLV: 22	Cooked: 22	—	—	—	14	—	—	—

Results of the following studies were not reported: References [43] and [56]—final set of FBRs was provided as daily cost of modeled diet, and daily or weekly consumption quantities are not reported. In reference [10], a basic food basket (fully nutritious and cost-effective) and 2 other food baskets considering dietary diversification strategies have been modeled. Only the FBR for basic food basket was reported in the Table.

Abbreviations: ASF, animal-source foods; DGLV, dark green leafy vegetable; FBR, food-based recommendation; NPNL, nonpregnant nonlactating; SSB, sugar-sweetened beverages.

**TABLE 5:** Summary of the final set of locally appropriate food-based recommendations included in the study for 6–23-mo-old children.

Country (study)	Age group (unit in food group recommendation)	Food groups/subgroups [(g/d), (servings/d), or (servings/wk)]						
		Cereals	Tubers/other starch/roots	Fruits and vegetables	Legumes, nuts, and seeds	Meat, fish, and eggs	Milk/milk products	Other
East Africa								
Kenya [44]/*Kitui* county	6–11 mo (servings/d)	Fortified cereals: 1; miller flour: 1	—	GLV: 1	Legumes, meat, fish, eggs: 1	—	Heat: treated full: fat cow or goat milk: ≥3	Breastfeed on demand
	12–23 mo (servings/d)	Fortified cereals: 1; miller flour: 1	—	GLV: 1	Legumes: 2	Meat, fish, and eggs: every day; small fish: ≥3 times per week	Heat: treated full: fat cow or goat milk: ≥3	Breastfeed on demand
Kenya [44] 1	6–11 mo (servings/d)	Fortified cereals: ≥3/wk	—	GLV: 1	Bean flour: 1	Small fish: ≥5 times per week	Heat: treated full: fat cow or goat milk: ≥2	Breastfeed on demand
	12–23 mo (servings/d)	Fortified cereals: ≥4/wk	—	GLV ≥2	Legumes or bean flour: 1	Small fish: ≥5 times per week	Heat: treated full: fat cow or goat milk: ≥3	Breastfeed on demand
Kenya [46] 2	6–8 mo infants (servings/wk)	—	30	5 vitamin A–rich fruits	Legumes: 30	—	40	—
	9–11 mo (servings/wk)	20	50	—	Legumes: 30	—	65	—
	12–23 mo (servings/wk)	20	50	90	Legumes: 30	—	—	—
Ethiopia [55] 3	6–8 mo (servings/wk)	Fortified infant cereals: 7, Grains: 4	—	—	—	—	7	Breastfeed on demand
	9–11 mo (servings/wk)	Grains: 7	—	7	Legumes: 14	—	7	Breastfeed on demand
	12–23 mo (servings/wk)	Grains: 14	—	3–4	legumes: 14	eggs: 7	7	Breastfeed on demand
Ethiopia [27]	6–8 mo (g/d)	154		24	8	35	208	Fats and oils: 8, beverages: 34
	9–12 mo (g/d)	198		15	3	55	206	Fats and oils: 10, beverages: 19
	12–23 mo (g/d)	284		8	2	36	133	Fats and oils: 18, beverages: 22
Tanzania [29]	6–8 mo (g/d)	33.3		259.2	46.5	Meat and fish: 5.6	1.8	Breast milk: 660
	9–11 mo (g/d)	61.6		261.9	41.3	Meat and fish: 35.8	2.7	Breast milk: 616
	12–23 mo (g/d)	—		—	—	—	—	—
Uganda [59]	12–23 mo (servings/wk) FBR1	—	Sweet potatoes: 7	Fruits: 14; GLV: 4	14	—	14	—
	12–23 mo (servings/wk) FBR2	Whole grain cereals: 14	Sweet potatoes: 7	GLV: 4	14	7	—	—
West Africa								
Ghana [28]	6–8 mo (servings/d)	—	—	3	Nuts and seeds: 1, beans: 1, soybean 1	—	1	Breastfeed on demand
	9–11 mo (servings/d)	Whole grain: 3	—	Fruits: 1; DGLV: 2	Beans: 1	Fish: 3	—	—
	12–23 mo breastfeeding (servings/d)	Whole grain: 1	—	DGLV: 2	Nuts and seeds: 3, beans: 1, cowpea: 1	—	1	—
	12–23 nonbreast feeding (servings/d)	Whole grain: 1	—	DGLV: 2	Nuts and seeds: 3, beans: 1, cowpea: 1	—	—	—

Results of the following studies were not reported: References [43] and [56]—final set of FBRs was provided as daily cost of modeled diet, and daily or weekly consumption quantities are not reported; Reference [45] reported the same set of FBRs developed in Reference [46] as their primary objective was to identify the factors opposing implementation of FBRs. Therefore, we did not report the FBRs in Reference [45] to avoid repetition of same results; Reference [58] reported the final set of FBRs in terms of 14 food items. We did not report the FBRs in the table to maintain the consistency. For the following studies, part of the results were reported: References [44], [46], and [55].

Abbreviations: ASF, animal-source food; DGLV, dark green leafy vegetable; FBR, food-based recommendation; GLV, green leafy vegetable; SSB, sugar-sweetened beverages.

1 Reference [44] reported 4 sets of FBRs for 2 age groups in 2 different areas in Kenya. FBRs provided for 1 age group are reported in this table.

2 Reference [46] modeled diets for 3 age groups of infants and young children across 3 livelihood groups in Northern Kenya (settled communities/Pastoralist communities, and Agro-pastoralist communities). Only the FBRs for 3 age groups in settled communities are reported in this table.

3 Reference [55] modeled diets of 3 age groups in 4 different regions in Ethiopia. Only the FBRs developed for 3 age groups in Tigray region are reported in this table.

## Discussion

### LP as a tool for diet optimization

LP is one of the successful optimization models in developing food patterns and providing valuable insights into the development of FBDGs and related nutritional policies. It identifies the most cost-effective combination of locally available foods to meet dietary needs and is adaptable in integrating various constraints, such as nutrient requirements, food preferences, and cost considerations. This adaptability is particularly critical in SSA, where dietary diversity is often restricted, and the affordability of nutrient-dense foods presents a significant challenge. As FBRs derived from this approach are based on actual dietary patterns and their costs, the recommended food is assumed to be available, affordable, and acceptable for the target population [12].

The primary objective of the reviewed studies is to construct nutritionally adequate and economically feasible dietary patterns, rather than to address multiple chronic nutrition-related conditions concurrently. This distinction underscores the differing priorities between low-resource and high-resource settings in dietary modeling efforts. In resource-constrained contexts such as SSA, the emphasis is predominantly on achieving micronutrient adequacy and enhancing food access as a means of addressing widespread malnutrition and food insecurity. Consequently, these studies typically prioritize cost-effective and context-appropriate strategies aimed at closing nutrient gaps, rather than focusing on the prevention of chronic diseases. Conversely, in resource-rich settings, where food availability, diversity, and access are less pressing concerns, dietary modeling efforts are more likely to center on chronic disease prevention and the promotion of sustainable, health-supportive dietary patterns. Notably, a small but emerging body of work in low-resource settings has begun to integrate chronic disease prevention into LP models by incorporating recommended nutrient intakes for preventing chronic diseases as LP model constraints. For instance, studies by Levesque et al. [17] and Azupogo et al. [20], conducted within the frameworks of national FBDGs development in Benin and Ghana, respectively, exemplify this evolving approach. These contributions highlight an emerging yet important shift toward aligning immediate nutritional adequacy with longer-term health promotion objectives in resource-limited environments. Darmon et al. [2] and Ferguson et al. [12] demonstrated that LP can transform local food consumption data into actionable dietary recommendations in Malawi. Parlesak et al. [10] in Mozambique and Levesque et al. [17] in Benin illustrated that LP models can support the creation of realistic, nutritionally complete food baskets aligned with local food availability. Furthermore, Termote et al. [43] and Talsma et al. [50] evaluated the contributions of biodiversity and biofortified crops to diet quality in Kenya. These studies demonstrate how LP can be extended beyond conventional food-based solutions to incorporate innovative approaches.

When food is available, the main obstacle is often economic; people may be unable to afford a nutritious diet even if they know what food to eat [68]. This is a critical consideration for policymakers seeking to sustainably improve nutrition. LP offers a framework for developing cost-effective and locally relevant dietary policies. Studies in Ethiopia [55,60] and Zambia [57] have applied LP to design nutrient-adequate diets that meet daily requirements although remaining affordable. Parlesak et al. [10] demonstrated how LP can optimize the inclusion of nutrient-dense foods in household diets, contributing to policy discussions on diet diversification.

Although LP models provide optimized dietary solutions, cultural preferences may hinder their implementation. Ferguson et al. [44] and Hotz et al. [45] expanded LP to rural Kenya, underscoring the benefits of combining LP with qualitative methods for enhanced contextual relevance. Hotz et al. [45] and Vossenaar et al. [46] in Kenya, as well as Wessells et al. [54] in Niger, integrated LP analysis with focused ethnographic studies to ensure that the derived FBRs align with local food preferences and social norms. This is important in developing context-specific food-based nutrition intervention strategies to support implementation of the FBRs. LP analysis can also strengthen linkages between agriculture and nutrition by identifying nutrient-dense local crops that, when cultivated on a larger scale, can improve dietary quality. Recent studies have expanded LP applications by incorporating environmental sustainability considerations and refining the specificity of model inputs. Azupogo et al. [20] formulated healthier and more environmentally sustainable diets in Ghana.

The growing use of LP in SSA is strongly supported by the availability of user-friendly software, such as Optifood. These tools make the methodology more accessible to public health practitioners and have been successfully used in various settings to formulate practical, evidence-based recommendations. This has significantly contributed to initiatives aimed at combating malnutrition and promoting sustainable improvements in dietary practices [69].

Nevertheless, many authors caution that LP results depend heavily on the accuracy and quality of the input parameters, particularly food composition data and nutrient requirement estimates. Studies such as Berra [27] in Ethiopia and Sayed and Schönfeldt [58] in South Africa note that LP is sensitive to model assumptions and the choice of model parameters, which must be rigorously justified to ensure reliable outputs. For example, in most LP models, parameters such as decision variables (typically represented by the quantities of food items), along with acceptability constraints (defined by the upper and lower bounds of food consumption distributions within the target population) are generally derived from 24-h dietary recall data. These dietary recalls are commonly collected for a single day per individual, or as averages from multiple nonconsecutive or consecutive 24-h periods. This is widely adopted, particularly in low-income and middle-income countries with no exception in SSA, where repeated dietary assessments are often constrained by limited resources and logistical challenges. Despite its practicality, this approach is subject to both random and systematic errors [70]. These include participant recall bias, interviewer bias, and inaccuracies in portion size estimation [28]. Consequently, this may result in either an underestimation of nutrient inadequacies or an overestimation of nutrient adequacy within the population. Some studies used data from FAO food balance sheets [42,53] and national food consumption surveys [20,62], resulting in dietary patterns that are representative at the national level. However, such diets may not accurately capture the nutritional needs or dietary preferences of specific population subgroups or account for regional variations. Nevertheless, these national-level diets often serve as a foundational reference for the development of more targeted dietary models.

Some studies developed food inventories based on market surveys and commonly consumed food items identified through secondary data sources [10], rather than relying on primary 24-h dietary recall data. However, the findings presented in the reviewed studies clearly indicate that the outcomes are heavily influenced by the researchers’ choices of the most suitable micronutrient-rich, affordable, and locally accessible food items [10,43]. Similarly, when conducting market surveys to determine food costs for the purpose of identifying a minimum cost-optimized diet, the methodology of data collection is critical. Factors such as the type of price data (e.g., retail prices), as well as the geographic location and timing of data collection, significantly influence the reliability and representativeness of the cost estimates.

Moreover, many studies rely on cross-sectional data, which, by design, capture dietary intake at a single point in time and may not adequately reflect seasonal fluctuations in food availability or consumption patterns [61]. This limitation is particularly salient in SSA, a region highly susceptible to weather-related shocks. Such climatic variability not only affects agricultural productivity and market prices but also contributes to seasonal volatility in food prices [71], thereby influencing dietary choices across different times of the year. Despite the inherent constraints of cross-sectional data, some studies have sought to address this issue by developing FBRs tailored to specific seasons—such as the harvest and lean periods—thus incorporating an element of seasonality into dietary planning [2,12].

Once the food list is prepared, the nutrient composition of food items was generated using various national and regional food composition tables. In the absence of national databases, researchers relied on tables from other countries for instance West African Food Composition Table [20], which may introduce inaccuracies. Nutrient content can vary significantly due to regional factors such as farming practices, and food processing and these variations can affect the reliability of study outcomes, especially if the data do not accurately reflect the nutrient composition of locally consumed foods [72].

### Proven value of optimization in providing FBRs

SSA currently has the lowest proportion of its population meeting internationally recognized dietary quality standards [73]. Many African diets not only reflect inadequate supplies of dietary energy but also exhibit deficiencies in essential nutrients, such as iron and vitamin A, even among individuals achieving energy sufficiency [74]. Addressing these issues requires effective intervention regarding diet quality. Among the available strategies, food-based dietary interventions are the most sustainable, provided that nutritionally adequate diets based on locally available foods can be identified and effectively promoted [12].

Despite certain limitations, FBDGs are widely regarded as invaluable tools for promoting healthy eating habits [75]. Many African countries currently lack these guidelines, with only 11 (Benin, Ethiopia, Gabon, Ghana, Kenya, Namibia, Nigeria, Seychelles, Sierra Leone, Zambia, and South Africa) of 48 countries having developed country-specific FBDGs [40]. In the absence of officially endorsed national dietary guidelines, the formulation of context-specific FBRs has been instrumental in improving diets and, consequently, nutritional status [76]. In the SSA region, prioritizing the development of new FBDGs or updating existing ones is imperative [77].

Developing FBDGs poses significant challenges. Evaluating habitual dietary intake requires accounting for variations within and between individuals [55]. Additionally, regional differences in food availability and consumption patterns necessitate separate dietary guidelines for different areas [55]. Therefore, traditional methods for developing FBDGs often rely on extensive consultations, which can be time consuming, cumbersome, and prone to bias [12].

**FIGURE 4:**
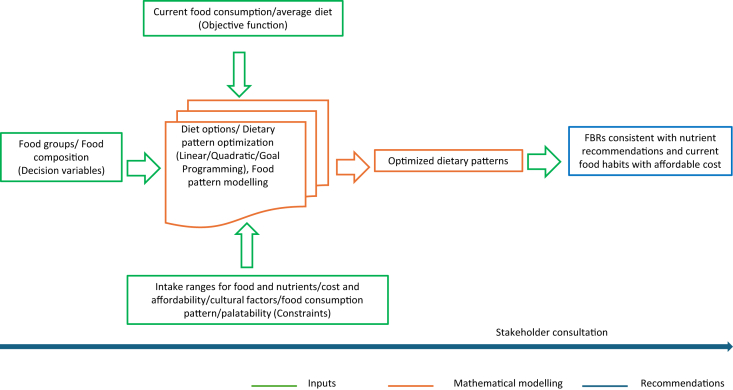
Overview of the development process for food based dietary guidelines from diet optimization using mathematical modeling. Source: complied using reviewed literature [13,19,76].

An alternative and more efficient approach involves computer-based modeling to minimize these challenges and enhance the accuracy and objectivity of guidelines [12]. Diet modeling formulates recommended daily dietary patterns that align with these objectives while considering additional factors such as acceptability and cost. These guidelines can serve as tools for nutrition education or target setting—for example, Ethiopia [60] and Ghana [20]. The reviewed studies highlight the value of diet optimization in developing evidence-based, feasible dietary guidelines and interventions in various settings across SSA. Figure 4 [13,19,76] illustrates the systematic progression of the FBRs and FBDGs development process using mathematical modeling.

Although effective, LP has several limitations that warrant discussion. First, the success of LP models depends on the availability and accuracy of local food composition data. In regions where data are sparse or outdated, the reliability of LP model outputs may be compromised. Second, LP alone may not fully address the behavioral and practical aspects of food consumption. Without considering these aspects, practical implementation and adherence to optimized diets may prove challenging, underscoring the need for interdisciplinary approaches. Third, LP theory presents several open challenges, including the pursuit of strong polynomial-time performance relative to the number of constraints and variables [3]. Furthermore, LP is not applicable to multiple objective functions, necessitating the adoption of multiobjective decision-making methods such as goal programming [26].

Overall, the reviewed studies highlight the versatility and value of LP as a methodological approach in diet modeling. By combining rigor, adaptability, and cost-effectiveness, LP analysis has become a cornerstone for addressing dietary and nutritional challenges in SSA.

### Research and policy recommendations

We propose the following recommendations:

1. Promote context-specific dietary modeling. Policymakers and researchers should prioritize the use of mathematical programming, such as LP, to develop FBRs tailored to the regional context of SSA. This entails incorporating locally available foods, traditional dietary patterns, and region-specific nutrient deficiencies to ensure feasible and culturally acceptable interventions.
2. Enhance data collection quality. Reliable and comprehensive data sets on local food systems, nutrient compositions, and consumption patterns are essential for effective diet modeling. Governments and development partners should invest in improving food-related data systems in SSA countries to ensure accurate and actionable mathematical programming.
3. Integrate interdisciplinary collaboration. The complexity of nutrition modeling demands inputs from nutritionists, economists, data scientists, and social scientists to address both technical and contextual challenges of dietary modeling. Establishing multidisciplinary research networks and fostering collaboration can enhance the applicability of diet optimization methods in SSA.
4. Expand capacity building for mathematical optimization tools. Governments and institutions should strengthen local capacity in mathematical optimization techniques for diet modeling. This includes training researchers, practitioners, and policymakers in using and interpreting outputs from LP models and other relevant tools, particularly in resource-constrained settings.
5. Facilitate policy uptake and implementation. To ensure the transition toward healthier diets, strong policy support is essential for translating model-derived dietary recommendations into actionable programs. Governments should integrate the findings from mathematical programming studies into national nutrition strategies, focusing on implementation through school feeding programs, health care systems, and community-based nutrition initiatives.
6. Monitor and evaluate the impact of optimized FBRs in a real-world setting. We recognize that limited research has been conducted to evaluate the effectiveness of these FBRs through direct application in real-world settings. The lack of such evaluations highlights a critical research gap that needs to be addressed.

## Conclusions

This study provides a scoping review of how mathematical modeling is used to formulate nutritionally adequate, culturally relevant, and cost-effective dietary solutions in SSA. It also identifies opportunities to refine LP-based approaches, ensuring that they remain vital tools for addressing global nutritional challenges. The growing body of literature underscores the value of LP as a robust method for dietary modeling, particularly in resource-constrained settings. Iterative LP can develop FBRs that align with local food systems while addressing specific nutrient deficiencies. Given the complexity of nutrition modeling and the sociocultural context of food consumption, careful consideration of input data and interdisciplinary collaboration on the implementation of LP-generated dietary recommendations is essential.

## Author contributions

The authors’ responsibilities were as follows—SS: was responsible for conceptualization, methodology, validation, investigation, project administration, writing (review and editing), and funding acquisition; MGDA: contributed to methodology, formal analysis, data curation, visualization, and writing (original draft); and both authors: have read and approved the final manuscript.

## Data availability

This study is a scoping review that used only publicly accessible documents.

## Declaration of generative AI and AI-assisted technologies in the writing process

During the preparation of this work, the authors used Paperpal to check grammar. After using this tool, the authors reviewed and edited the content as needed and take full responsibility for the final publication.

## Funding

This study was financially supported by JSPS KAKENHI Grant Number JP19KK0341 and the Foundation for Dietary Scientific Research (special research grant). These sponsors had no role in the design, analysis, or writing of this article.

## Conflict of interest

SS reports financial support by Japan Society for the Promotion of Science and article publishing charges by the Foundation for Dietary Scientific Research. All other authors report no conflicts of interest.
