# Supplementary material for: Relevance of Mathematical Optimization as a Tool for Diet Modeling in the Development of Food-Based Dietary Recommendations in Sub-Saharan Africa: A Scoping Review
Source: Adv Nutr. 2025 Jul 11;16(8):100480. doi: 10.1016/j.advnut.2025.100480 (PMC12335993; doi:10.1016/j.advnut.2025.100480)
Supplement: Multimedia component 1 [file mmc1.docx]

**“Relevance of mathematical optimization as a tool for diet modelling in the development of food-based dietary recommendations in Sub-Saharan Africa: A scoping review”**

Sakiko Shiratori and MG Dilini Abeysekara

Supplementary Material: research strategy

# Web of Science Search Strategy (v0.1) # Database: Web of Science Core Collection # Entitlements:

- WOS.SCI: 1900 to 2025
- WOS.BHCI: 2005 to 2025
- WOS.BSCI: 2005 to 2025
- WOS.ESCI: 2005 to 2025 - WOS.SSCI: 1956 to 2025

# Searches:

1: ((((((((((((((((((ALL=("food based recommendations")) OR ALL=("dietary recommendations"))

OR ALL=("sustainable diet")) OR ALL=("EAT Lancet diet")) OR ALL=("planetary health diet")) OR ALL=(" healthy and sustainable diet"))OR ALL=("healthy diet")) OR ALL=("nutritional guidelines")) OR ALL=("dietary guidelines")) OR ALL=("food pyramid")) OR ALL=("food basket")) OR ALL=("low cost diet")) OR ALL=("eating plate"))OR ALL=("recipe")) OR ALL=("meal")) OR ALL=("balanced diet")) OR ALL=("ideal diet")) OR ALL=("adequate diet")) OR ALL= ("optimal diet") AND (LA==("ENGLISH")) Timespan: 2000-01-01 to 2024-05-31 Date Run: Fri May 16

2025 09:37:18 GMT+0900 (Japan Standard Time) Results: 116093

2: ((((((((((ALL=("diet modelling")) OR ALL=("diet optimization")) OR ALL=("mathematical programming")) OR ALL=("linear programming")) OR ALL=("quadratic programming")) OR ALL=("goal programming")) OR ALL=("cost minimization")) OR ALL=("optifood")) OR ALL=("cost of diet")) OR ALL=("nutrient optimization")) AND (LA==("ENGLISH")) Timespan: 2000-01-01 to 2024-05-31 Date Run: Fri May 16 2025 09:39:22 GMT+0900 (Japan Standard

Time) Results: 66175

3: (((((((((((((((((((((((((((((((((((((((((((((((((((((ALL= ("Africa*")) OR ALL= ("Sub-Sahara*")) OR

ALL=("Sub Sahara*")) OR ALL=(SSA)) OR ALL= ("Sahel*")) OR ALL=(Angola)) OR

ALL=(Benin)) OR ALL=(Botswana)) OR ALL=("Burkina Faso")) OR ALL=(Burundi)) OR

ALL=("Cape verde")) OR ALL=(Cameroon)) OR ALL=("Central African Republic")) OR

ALL=(Chad)) OR ALL=(Comoros)) OR ALL=("Democratic Republic of Congo")) OR

ALL=("Republic of Congo")) OR ALL=(“Cote d’Ivoire”)) OR ALL=("Equatorial Guinea")) OR

ALL=(Eritrea)) OR ALL=(Eswatini)) OR ALL=(Ethiopia)) OR ALL=(Gabon)) OR ALL=(Gambia))

OR ALL=(Ghana)) OR ALL=(Guinea)) OR ALL=("Guinea Bissau")) OR ALL=(Kenya)) OR

ALL=(Lesotho)) OR ALL=(Liberia)) OR ALL=(Madagascar)) OR ALL=(Malawi)) OR

ALL=(Mali))OR ALL=(Mauritania)) OR ALL=(Mauritius)) OR ALL=(Mozambique)) OR

ALL=(Namibia)) OR

ALL=(Niger)) OR ALL=(Nigeria)) OR ALL=(Rwanda)) OR ALL=("Sao Tome")) OR ALL=("Sao

Tome and Principe")) OR ALL=(Senegal)) OR ALL=(Seychelles)) OR ALL=("Sierra Leone")) OR

ALL=(Somalia)) OR ALL=("South Sudan")) OR ALL =(Sudan)) OR ALL=(Tanzania)) OR

ALL=(Togo)) OR ALL=(Uganda)) OR ALL=(Zambia)) OR ALL= (Zimbabwe)) AND

(LA==("English")) Timespan: 2000-01-01 to 2024-05-31 Date Run: Fri May 16 2025 09:41:46

GMT+0900 (Japan Standard Time) Results: 1540592

4: #1 AND #2 AND #3 Date Run: Fri May 16 2025 09:42:31 GMT+0900 (Japan Standard Time) Results: 45

5: #1 AND #3 Date Run: Fri May 16 2025 09:43:06 GMT+0900 (Japan Standard

Time) Results: 6537

6: #2 AND #3 Date Run: Fri May 16 2025 09:43:20 GMT+0900 (Japan Standard

Time) Results: 960
